# Supplementary material for: Resolving the full spectrum of human genome variation using Linked-Reads
Source: Genome Res. 2019 Apr;29(4):635–45. doi: 10.1101/gr.234443.118 (PMC6442396; doi:10.1101/gr.234443.118)
Supplement: Supplemental Material [file supp_gr.234443.118_Supplemental_longranger-2.2.zip › longranger-2.2.tar.bz2!/lr2.2-public/lib/stan/cmdstan/stan/doc/api/html/unit__e__point_8hpp_source.html]

Stan: src/stan/mcmc/hmc/hamiltonians/unit\_e\_point.hpp Source File


|  |  |
| --- | --- |
|  | Stan  2.5.0  probability, sampling & optimization |


- Main Page
- Related Pages
- Namespaces
- Classes
- Files

- File List
- File Members

All Classes Namespaces Files Functions Variables Typedefs Enumerations Enumerator Friends Macros Pages

- src
- stan
- mcmc
- hmc
- hamiltonians

unit\_e\_point.hpp

Go to the documentation of this file.

1 #ifndef STAN\_\_MCMC\_\_UNIT\_\_E\_\_POINT\_\_BETA

2 #define STAN\_\_MCMC\_\_UNIT\_\_E\_\_POINT\_\_BETA

3

4 #include <stan/mcmc/hmc/hamiltonians/ps\_point.hpp>

5

6 namespace stan {

7

8  namespace mcmc {

9

10  // Point in a phase space with a base

11  // Euclidean manifold with unit metric

12  class unit\_e\_point: public ps\_point {

13

14  public:

15

16  unit\_e\_point(int n): ps\_point(n) {};

17

18  };

19

20  } // mcmc

21

22 } // stan

23

24

25 #endif

stan::mcmc::ps\_point

**Definition:** ps\_point.hpp:16

stan::mcmc::unit\_e\_point::unit\_e\_point

unit\_e\_point(int n)

**Definition:** unit\_e\_point.hpp:16

stan::mcmc::unit\_e\_point

**Definition:** unit\_e\_point.hpp:12

ps\_point.hpp


---

[ Stan Home Page ]

*© 2011–2014,
Stan Development Team.*
